# Containing influenza outbreaks with antiviral use in long‐term care facilities in Taiwan, 2008‐2014

**DOI:** 10.1111/irv.12536

**Published:** 2018-01-28

**Authors:** Hao‐Yuan Cheng, Wan‐Chin Chen, Yu‐Ju Chou, Angela Song‐En Huang, Wan‐Ting Huang

**Affiliations:** ^1^ Epidemic Intelligence Center Taiwan Centers for Disease Control Taipei Taiwan; ^2^ Office of Preventive Medicine Taiwan Centers for Disease Control Taipei Taiwan; ^3^ Division of Acute Infectious Diseases Taiwan Centers for Disease Control Taipei Taiwan

**Keywords:** antivirals, influenza outbreak, long‐term care facilities, prophylaxis

## Abstract

**Backgrounds:**

Influenza can spread rapidly in long‐term care facilities (LTCFs), and residents are usually at higher risk for influenza infections.

**Objective:**

Our study aimed to evaluate the effectiveness of antiviral interventions on outbreak control.

**Methods:**

Taiwan Centers for Disease Control used a syndromic surveillance system to monitor outbreaks in LTCFs. Local public health authorities verified those outbreaks and logged reports to the Epidemic Investigation Report Files Management System (EIRFMS). We conducted a retrospective cohort study by reviewing EIRFMS reports of influenza outbreaks in LTCFs during 2008‐2014. An influenza outbreak was defined as 3 or more cases of influenza‐like illness occurring within a 48‐hours period with ≥1 case of real‐time RT‐PCR‐confirmed influenza in the same LTCF. Antiviral interventions included providing antiviral treatment for patients and antiviral prophylaxis for contacts during outbreaks.

**Results:**

Of 102 influenza outbreaks, median days from onset of the first patient to outbreak notification was 4 (range 0‐22). Median attack rate was 24% (range 2.2%‐100%). Median influenza vaccination coverage among residents was 81% (range 0%‐100%); 43% occurred during the summer months. Even though antiviral treatment was provided in 87% of the outbreaks, antiviral prophylaxis was implemented in only 40%. Starting antiviral treatment within 2 days of outbreak onset was associated with keeping attack rates at <25% (OR 0.29, 95% CI: 0.12‐0.71).

**Conclusions:**

Early initiation of antiviral treatment may reduce the magnitude of influenza outbreaks. Clinicians should identify patients with influenza and start antiviral use early to prevent large outbreaks in LTCFs.

## 1 INTRODUCTION

Long‐term care facilities (LTCFs), including board and care home, assisted living facilities and nursing homes, provide health care to individuals who cannot independently take care of themselves. Residents living in these facilities are usually elderly or those with chronic diseases, such as cardiovascular disease, diabetes mellitus, or dementia, making the residents at high risk for influenza infection.^1^ Furthermore, influenza can spread rapidly in congregate settings.^2^ As a result, influenza outbreaks in LTCFs last longer, causing more severe complications, and leading to increased mortality.

There are 1058 LTCFs around Taiwan as of August 2016.^3^ Every year, 10‐15 influenza outbreaks in these facilities were reported. Previously, control of influenza outbreaks in LTCFs usually relied on non‐pharmaceutical interventions, including patient isolation, personal hygiene enhancement, and environmental disinfection. After the licensure of neuraminidase inhibitor, many guidelines started to recommend the use of antiviral interventions, including antiviral treatment and antiviral prophylaxis, for outbreak control.^4^, ^5^, ^6^ Although findings from a modeling analysis found that antiviral prophylaxis use was effective in controlling outbreaks, other studies, mainly observational, could not quantify the effectiveness of such interventions and make definitive conclusions.^5^, ^7^

In Taiwan, implementation of antiviral interventions in outbreaks increased gradually after the 2009 H1N1 influenza pandemic. Although the use of antiviral treatment for high‐risk groups, such as those with underlying diseases, the elderly and residents in LTCFs, has become standard clinical practice in Taiwan, no guidelines for antiviral use as part of outbreak control have been established yet. The initiation of antiviral prophylaxis depended on the on‐site judgment of public health practitioners and varied from outbreak to outbreak, making the evaluation of effectiveness infeasible.

Our study aimed to describe the epidemiological characteristics of influenza outbreaks in LTCFs in Taiwan and to evaluate the effectiveness of antiviral interventions on outbreak control.

## 2 METHOD**S**

### 2.1 Study design and data collection

Taiwan Centers for Disease Control (Taiwan CDC) used a syndromic surveillance system to monitor outbreaks in populous institutions, including LTCFs, around Taiwan. After outbreaks were notified with an influenza‐like illness (ILI), local public health practitioners must verify the outbreaks, submit appropriate patient specimens to the reference laboratory of Taiwan CDC for the identification of causative agents, and log the outbreak investigation reports to the Epidemic Investigation Report Files Management System (EIRFMS). ILI was defined as an acute respiratory infection (symptoms including cough) with fever and had at least one of the following symptoms: soreness, headache, or malaise. Influenza infection was then confirmed using real‐time reverse transcription‐polymerase chain reaction (RT‐PCR) test.

We conducted a retrospective cohort study and reviewed EIRFMS‐logged reports of influenza outbreaks in LTCFs during 2008‐2014. An influenza outbreak was defined as an incident with 3 or more patients having ILI occurring within a 48‐hours period and at least one patient having laboratory‐confirmed influenza infection in the same LTCF.^8^

Date of outbreak start, notification, public health response, source of infection, total number of persons affected, duration of outbreak, and dates that of antiviral treatment and prophylaxis began were extracted from the outbreak reports. Date of outbreak start referred to the symptom onset date of the first ILI patient, and duration of outbreak was measured as the elapsed time from outbreak start to the date of outbreak end, which was the symptom onset of the last patient. Antiviral treatment was defined as the therapeutic use of antiviral agents for symptomatic patient. Antiviral prophylaxis was defined as the prophylactic use for non‐ill contacts who lived in the same room or had daily activities in the same area as patients after the outbreak was notified. The antiviral agents used in an influenza outbreak were neuraminidase inhibitors, including oseltamivir (Tamiflu^®^) and zanamivir (Relenza^®^). The dosages of antiviral treatment and prophylaxis for adults and children were based on the US CDC recommendation.^9^ The regimen of antiviral treatment was one dose given twice a day for 5 days, and the regimen of antiviral prophylaxis was one dose given once a day for 10 days. Prophylaxis was given only once during each outbreak, even if subsequent cases occurred. A large outbreak was defined as an outbreak with attack rate ≥ 25% because the median attack rate of the influenza outbreaks during the study period was around 25%.

### 2.2 Statistical analysis

Descriptive analysis was performed to characterize the influenza outbreaks. Chi‐square test for categorical variables and Wilcoxon test for nonparametric continuous variables were used to identify factors associated with the magnitudes of outbreaks, such as viral etiology, the elapsed time from outbreak start to notification and response and the use of antiviral interventions.

In a multiple logistic regression model, starting antiviral treatment within 2 days of outbreak start and use of antiviral prophylaxis at least 2 days, the average incubation period of influenza, before date of outbreak end were evaluated, along with previously reported risk factors, including type of influenza virus^10^, ^11^, ^12^ and outbreak notification within 3 days,^13^ to clarify whether these interventions were independently associated with large outbreaks. Data were maintained in Microsoft Excel 2016 (Bellevue, WA) and analyzed using stata 12.0 (Stata Corp, College Station, TX).

### 2.3 Ethical approval

Because the data in outbreak reports were collected in response to the influenza outbreaks in those facilities, an ethical approval was waived.

## 3 RESULTS

**Figure 1:**
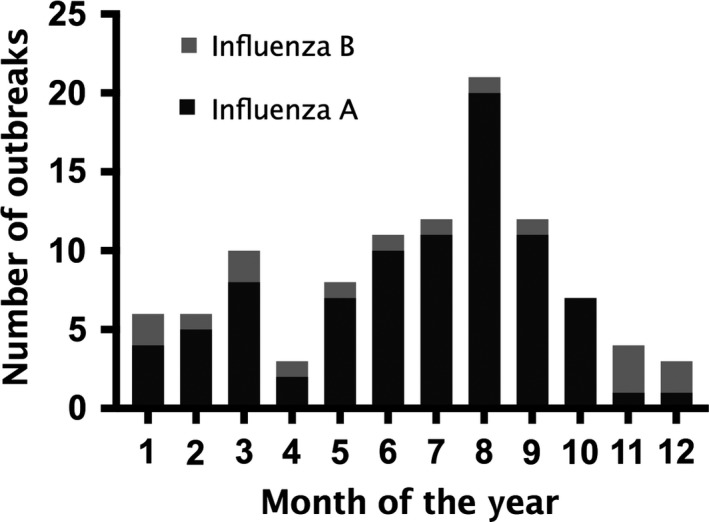
Monthly number of influenza outbreaks in long‐term care facilities, 2008‐2014

A total of 102 influenza outbreaks were logged during the 7‐year period, with outbreaks involving a median of 12 patients (range 3‐75). The median number of residents in the facilities was 65.5 (11‐402). Median days from outbreak start to outbreak notification was 4 (range 0‐22 days). Median attack rate was 24% (range 2.2%‐100%). Of the 102 outbreaks, 87% were caused by influenza A; of which, 84% (72 of 86) were H3N2. In 18% of the outbreaks, an affected staff in the facilities was the first patient with ILI. Outbreaks peaked in August, with 40% of the outbreaks occurring during the summer months (June, July, and August) (Figure 1).

Median influenza vaccination rate of residents is 81% (range 0%‐100%). Even though 87% of outbreaks used antiviral treatment, only 40% used antivirals for prophylaxis (Table 1). Median time elapsed from outbreak start to antiviral treatment was 3 days (range 0‐24) and from notification to treatment was one day (range 0‐21). Vaccine coverage, type of influenza, time elapsed to outbreak notification, and infection control measures for outbreak did not have any statistically significant effect on the magnitude of outbreaks. For large outbreaks, we observed a lower proportion of starting antiviral treatment within 2 days (40% vs 60%, *P *=* *.03), and more use of antiviral prophylaxis (53% vs 27%, *P *=* *.009) (Table 2). It, however, took a median of 6 days (range 1‐25) from outbreak start to implement antiviral prophylaxis and a median of 0 days (range ‐5‐15) for the outbreak to end in those outbreaks with antiviral prophylaxis.

**Table 1:** Characteristic of influenza outbreaks in Taiwan, 2008‐2014

Number of outbreaks	Overall	Outbreak with AR < 25% (n = 51)	Outbreak with AR ≥ 25% (n = 51)
Number of residents	65.5 (11‐402)	82 (18‐402)	58 (11‐279)
Number of patients (persons)	12 (3‐75)	9 (3‐48)	19 (5‐75)
Vaccine coverage (%)	79 (0‐100)	83 (39‐100)	76 (0‐100)
Outbreak duration (d)	8 (0‐26)	6 (0‐20)	8 (2‐26)
Attack rate (%)	24 (2.2‐100)	11.0 (2.2‐23.0)	33.5 (25‐100)
Days from outbreak start to notification	4 (0‐23)	5 (0‐23)	4 (1‐22)
Notification within 3 d (%)	38 (37)	17 (33)	21 (41)
Days from outbreak start to public health response	5 (1‐24)	5 (1‐24)	4 (1‐22)
Etiology (%)			
Influenza A	86 (84)	41 (80)	45 (88)
Influenza B	16 (16)	10 (20)	6 (12)
First symptomatic patient of outbreak (%)			
Staff	18 (18)	6 (12)	12 (24)
Residents	84 (82)	45 (88)	39 (76)
Antiviral intervention for outbreak (%)			
Treatment	89 (87)	44 (86)	45 (88)
Treatment started within 2 d after outbreak start^a^	53 (52)	32 (60)	21 (40)
Prophylaxis^b^	41 (40)	14 (27)	27 (53)
Prophylaxis started within 2 d after outbreak start	2 (2)	1 (1)	1 (1)
Prophylaxis started at least 2 d before date of outbreak end^c^	17 (17)	4 (8)	13 (26)

AR, attack rate.

Results are expressed as n (%) or median (range).

a *P* value, .03.

b *P* value, .009.

c *P* value, .017.

**Table 2:** Univariate and multivariate analysis of factors associated with being a large influenza outbreak in long‐term care facilities in Taiwan, 2008‐2014

	Outbreak with AR < 25% (n = 51), %	Outbreak with AR ≥ 25% (n = 51), %	Crude OR	95% CI	Adjusted OR	95% CI
Notification within 3 d	17 (33)	21 (41)	1.40	0.63‐3.13	2.03	0.82‐5.00
Etiology						
Influenza A	41 (80)	45 (88)	1.0	–	1.0	–
Influenza B	10 (20)	6 (12)	0.55	0.18‐1.64	0.49	0.22‐3.38
Antiviral treatment						
Within 2 d after outbreak start	32 (60)	21 (40)	0.42	0.19‐0.92	0.30	0.12‐0.72
Antiviral prophylaxis						
Started at least 2 d before date of outbreak end	4 (8)	13 (26)	4.02	1.53‐1.23	5.26	1.46‐19.98

AR, attack rate; OR, odds ratio; CI, confidence interval.

Results are expressed as n (%).

In multivariate analyses, initiating antiviral treatment within 2 days of outbreak start independently decreased the possibility of a large influenza outbreak to only one‐third in LTCFs (odds ratio [OR] 0.29, 95% confidence interval [CI] 0.12‐0.71). Although antiviral prophylaxis was an independent factor associated with a large outbreak (OR 3.81, 95% CI 1.55‐9.37), the effectiveness and causality could not be accurately estimated because it was usually started late in an outbreak (Table 2).

## 4 DISCUSSION

In this study, we analyze the epidemiological data of influenza outbreaks in LTCFs from 2008 to 2014. The effectiveness of antiviral prophylaxis could not be accurately estimated because it was usually started late in an outbreak, but early use of antiviral treatment may help to prevent a large influenza outbreak by decreasing the chance to only one‐third.

Influenza outbreaks in LTCFs in Taiwan mostly occurred during the summer months according to our analysis. Despite having a subtropical climate with mild winters, Taiwan does experience seasonal influenza epidemics with cases rising in December, peaking in late January to early February. This epidemic pattern was similar during our study periods, from 2008 to 2014.^14^ Outbreaks of influenza in LTCFs are expected to also occur during this period, as reported by the Canadian study,^13^ which found more influenza outbreaks in the winter months. The unexpected finding of LTCFs reporting outbreaks during the summer might result from the protective effect of Taiwan's influenza vaccination program, which begins to vaccinate staff and residents with trivalent influenza vaccine every October, achieving high coverage rates in LTCFs. The high coverage may be because residents of LTCFs are less mobile, can be easily approached, and are targeted in Taiwan's seasonal influenza vaccination program. The higher‐than 80% vaccination coverage may contribute to less influenza outbreaks in winter, from December to February of the following year. Protection provided by vaccination, however, may start to wane 3‐4 months later and decrease to a nadir in the summer.^15^, ^16^ Therefore, influenza outbreaks were frequently observed in the summer, from June to August, as the virus continues to circulate in the community at that time. Another explanation is possible antigen drift of circulating influenza virus in summer, for example, a vaccine‐mismatched influenza B‐Yamagata strain in 2011 and a drifted H3N2 strain in 2012.^17^ Because providing additional vaccination in the summer is not practical, alternative control measures, such as antiviral interventions, should be implemented when influenza outbreaks occur.

Our study found that early use of antiviral treatment was associated with a decreased possibility of large influenza outbreak in LTCFs. Previous observations also supported this finding by showing the impact of early treatment in influenza outbreak control.^13^, ^18^, ^19^, ^20^, ^21^ Although the clinical effectiveness of antiviral treatment in patients with influenza has been debated for a long time,^22^ use of antiviral agents in public health, for example, containing influenza outbreaks, may still be of value. Administration of oseltamivir could shorten the duration of clinical disease, decrease viral loads, and stop nasopharyngeal virus shedding soon after patients receive antiviral treatments.^23^, ^24^ A study conducted in Bangladesh also proved that treatment with oseltamivir could reduce the risk of transmitting influenza virus to another member in the same household.^25^ These features suggest the impacts of antiviral treatment use on outbreak control in populous institutions, because decreased duration of clinical symptoms or virus shedding could reduce the possibility of secondary transmission and therefore decrease the magnitude of an outbreak. In our study, the odds of development into a large outbreak could be reduced to only one‐third by early initiating antiviral treatment.

Although antiviral prophylaxis has also been recommended in many guidelines for controlling influenza outbreak,^2^, ^4^, ^19^ there were concerns about increase in drug resistance and tremendous costs of antiviral agents. In Taiwan, there is no guidance on using antiviral prophylaxis in influenza outbreaks in LTCFs. The decision was usually made on a case‐by‐case basis by Taiwan CDC, the agency that stockpiled the antiviral agents for influenza, after an outbreak was notified. Considerations on whether to initiate an antiviral prophylaxis included the timing, duration and attack rate at notification, vaccination coverage in residents, total cost of prophylaxis, risks for severe influenza infection among residents, and possibly induced drug resistance. According to Taiwan's laboratory surveillance data, the proportion of oseltamivir‐resistant influenza viruses was less than 1% of isolates tested in these years.^26^, ^27^ Although oseltamivir‐resistant influenza virus had been rarely reported in Taiwan since 2009, antiviral prophylaxis was usually delayed because of late notification or the reluctance of massive antiviral use with enormous cost.

While previous modeling study has demonstrated the effectiveness of antiviral prophylaxis in outbreak control,^7^ our study revealed sometimes the prophylaxis started on the last day of an outbreak, suggesting the outbreaks were more likely to end by itself because the average incubation period of influenza was about 2 days. In our study, more antiviral prophylaxis was implemented in large outbreaks with an attack rate ≥ 25%. This reverse correlation does not weaken the effectiveness of antiviral prophylaxis; instead, it points out that prophylaxis is usually considered only when an unmitigated outbreak reached high attack rate. Further cost‐effectiveness study can help to figure out whether early use of antiviral prophylaxis could actually reduce total cost of outbreak control and medical treatment, and thus encourages prophylaxis in early stage of an outbreak. On the other hand, early treatment strategy may be a more cost‐effective alternative because it may decrease the necessity of massive antiviral prophylaxis and associated costs.

There are limitations in our study. First, this study is observational rather than interventional. It was difficult to control all the confounding parameters besides the use of antiviral interventions, for example, the awareness of influenza outbreak or the degree of implementation of non‐pharmaceutical interventions. However, Taiwan CDC has developed the infection control protocol for LTCFs. The protocol provides guidance on how to maintain the syndromic surveillance system in LTCFs and how to follow the guidelines of non‐pharmaceutical control measures, such as the use of appropriate personal protection equipment and maintaining environmental hygiene. Taiwan CDC also conducts regular checkup and on‐job professional education for those healthcare workers in LTCFs to improve their awareness and knowledge. Therefore, we expected the differences among different facilities, such as the preparedness for outbreak, awareness, and capability of healthcare workers and clinicians, could be minimized. Furthermore, the notification and registration of an outbreak relied on local health workers to log in EIRFMS. As a result, the completeness may be questionable and the total number of outbreaks may be underestimated. However, the effect may be minimal because preventing outbreaks from occurring in LTCFs is an important mission of local health departments, and Regional Centers of Taiwan CDC supervise outbreak control and ensure all outbreaks logged after each notification. Lastly, most antiviral prophylaxis was started late in an outbreak; therefore, it is difficult to distinguish whether the outbreak subsided by naturally or through the use of prophylaxis. Further interventional study using standard protocol for antiviral prophylaxis may be required to confirm its effectiveness.

In summary, our study revealed antiviral intervention affect the control of influenza outbreaks in LTCFs and early initiation of antiviral treatment could reduce the risk of development into large outbreaks to about one‐third. Early notification of influenza outbreaks in LTCFs and increased awareness of antiviral role in containing outbreaks among clinicians could facilitate timely interventions.

## CONFLICT OF INTEREST

All authors report no conflict of interests relevant to this article.

## DISCLAIMER

This publication was funded in part by the Skoll Global Threats Fund (SGTF), through TEPHINET, a program of the Task Force for Global Health, Inc. Its contents are solely the responsibility of the authors and do not necessarily represent the views of The Task Force for Global Health, Inc., TEPHINET, or SGTF.
